# Disruption of the medical curriculum in a developing country: The need for medical students to be stronger than ever

**DOI:** 10.15694/mep.2020.000188.1

**Published:** 2020-09-03

**Authors:** Wafaa Mowlabaccus, Adnaan Mowlabaccus

**Affiliations:** 1 University of Mauritius

**Keywords:** final year medical students, COVID-19, developing country

## Abstract

This article was migrated. The article was marked as recommended.

Being a medical student from a low to moderate income country comes with its challenges particularly in moments where the world is facing with an uncharted territory; the COVID-19 pandemic. With no face to face interactions with lecturers, halt of clinical postings and uncertainty of when exams will be held, final year medical students are facing an unprecedented situation. Being a final year student, this personal view is aimed at describing the current situation of medical students in Mauritius, the numerous challenges that are being encountered and the coping strategies being adopted.

## Introduction

Mauritius, a small island in the Indian Ocean, has been under lockdown since early March 2020 with a total of 334 confirmed cases and 10 deaths ( Ministry of Health and Wellness, 2020). While the curfew is now being lifted in a phased manner in Mauritius, university will resume only in July 2020.

## Current situation of final year medical students

We are currently being faced with an uncertainty regarding the completion of a very long study and the inability to achieve clinical competencies which can be very overwhelming. Firstly, final year medical students are required to complete 10 months of clinical rotations to be allowed to sit for the final comprehensive exams. Unfortunately, the lockdown has caused interruptions in clinical postings. Reasons behind the prohibition of going to clinical placements are mainly to minimize interactions and thus helping to contain the spread of the virus and to protect medical students from exposing themselves. Secondly, no formal online webinars are being held for penultimate year students since final year is supposed to be exclusively for clerkships. Hence we are left on our own to revise for the upcoming exams.

## Challenges encountered

Being pulled from clinical rotations, we have not interacted with patients for more than 2 months now. There is a lurking fear that we might lose our touch for history taking and clinical examinations; two competencies that are crucial for a future physician. While it has been seen that new methodologies such as the ‘virtual check-in tool’ are being adopted by developed countries to transition to virtual medical curriculum, our university has limited resources to do so ( Johnston *et al.*, 2020). Uncertainty pertaining to upcoming exams is another challenge we are currently facing. Many universities elsewhere have deployed means to assess final year medical students. For instance, the Imperial College of London delivered their final year exams remotely during the pandemic. While this has been possible in the UK, the implementation of online exams might pose a problem in Mauritius considering the number of students who struggle to have access to fast internet. Finally, Mauritius being highly dependent on tourism, will be facing with devastating economic consequences. Many individuals are likely to lose their jobs including parents of many students. Financial instability of many medical students will most likely have a deleterious impact on not only their mental health but also on their education.

## Mental well-being of students and role of educators

As per se ‘future front liners’ we are expected to be resilient and proactive. However, with the global crisis persisting, our mental health is vulnerable. Scant support is currently being provided by the university to help penultimate students who are having difficult times coping with this crisis. Being left without adequate support from the university can exacerbate anxiety, stress and even depression among medical students. It is thus essential that medical students are not left on their own to deal with these unfavorable emotions but rather to have educators who can validate their emotions during nerve-wracking situations where there is a lot of uncertainty going on ( Wald, 2020). It is crucial for educators to support medical student’ wellbeing by creating flexible learning environment and having regular well-being webinars where students feel heard and acknowledged. We suggest an increase in communication between the university and medical students to help us through this difficult time. Moreover we advocate for the implementation of a ‘relationship-centered education’ with holistic humane teaching that is carried out in a dignified, respectful and inclusive learning environment ( Rabow, Newman and Remen, 2014).

## Coping stategies adopted

The way individuals cope with distressing situations vary. We believe that it is crucial that each student finds his or her own unique way to positively cope with the exceptional situation. For us, while waiting for the resumption of clinical rotations we are finding ways to replace the feeling of vulnerability with an impetus and new purpose. Reflective writing has been the major way that we have adopted to combat the invisible enemy. Alongside we are adopting a healthy diet, sleeping properly and doing at least 30 minutes of physical exercise per day.

## Conclusion

We, final year medical students are facing with a complex and unique situation. While it is our duty and moral responsibility to take care of ourselves, we are also counting on the unremitting support from our educators to help us through this difficult phase. As Viktor Frankl rightly said, ‘ *we don’t get to choose our difficulties, but we do have the freedom to select our responses.*’

## Take Home Messages

• As students from a developing country, we are uniquely being impacted by the pandemic.
• It is imperative that new strategies are adopted by educators to mitigate the effects of the global crisis.
• Tailored coping mechanisms should be adopted by each and every one.

## Notes On Contributors

Wafaa Mowlabaccus: Final medical student at the University of Mauritius. ORCID ID: https://orcid.org/0000-0003-3984-4203

Adnaan Mowlabaccus: Medical graduate from the University of Mauritius.
