# Supplementary material for: Disruption of the medical curriculum in a developing country: The need for medical students to be stronger than ever
Source: MedEdPublish (2016). 2020 Sep 3;9:188. [Version 1] doi: 10.15694/mep.2020.000188.1 (PMC10699364; doi:10.15694/mep.2020.000188.1)
Supplement: Peer review history

# Reviewer report: Reviewer response for version 1

**DOI:** 10.21956/mep.20130.r31068

**Published:** 2020-09-15

**Authors:** Trevor Gibbs

**Affiliations:** 1 AMEE

This review has been migrated. The reviewer awarded 5 stars out of 5 I really did enjoy reading this paper, given the importance of hearing the perspectives from the students , their uncertainty and how various universities / schools are responding to student need. We wrongly assume that the medical / health professions education playground is level- it is not, it is grossly un-level and it is clear that many students are not getting a good deal during Covid-19. Good to read about the coping strategies too. I would recommend this paper to all those involved with curriculum planning.

Reviewer Expertise:

NA

No decision status is available

# Reviewer report: Reviewer response for version 1

**DOI:** 10.21956/mep.20130.r31069

**Published:** 2020-09-11

**Authors:** Viktor Riklefs

**Affiliations:** 1 Karaganda Medical University

This review has been migrated. The reviewer awarded 5 stars out of 5 The author raises an important concern. Medical education is certainly never meant to be completely online, especially during the clerkships. One possible solution could be to postpone the exams and allow students to take the job under certain limitations and assign them clinical supervisors. Medicine is certainly a complex profession with serious requirements for continious education for lifetime. So the world needs to take now the proactive position and allow final year students who graduated with lack of training due to COVID-19 situation provide with enough opportunities to receive good clinical training in later phases of education. It would be interesting to see, how the situation resolved in Mauritius. This article certainly needs to be dessiminated further.

Reviewer Expertise:

NA

No decision status is available

# Reviewer report: Reviewer response for version 1

**DOI:** 10.21956/mep.20130.r31070

**Published:** 2020-09-04

**Authors:** P Ravi Shankar

**Affiliations:** 1 American International Medical University

This review has been migrated. The reviewer awarded 4 stars out of 5 This is an interesting perspective from students in a medical school in Mauritius. As has been mentioned in other articles in the journal the challenges for medical schools and students in developing nations are greater. Most developing nations had not put in place a system for online learning before the pandemic. Hence the abrupt shift to online learning may have been more challenging. Studies have shown final year students may be under greater stress as they are concerned about graduating on time and about their job and further education prospects. Uncertainty is a major challenge associated with the pandemic and as mentioned in other articles in MedEdPublish timely communication from the institution may be important in reducing anxiety.

Reviewer Expertise:

NA

No decision status is available
